# Understanding junior doctors’ experiences of teaching on the acute take: a qualitative study

**DOI:** 10.1186/s12909-021-02815-4

**Published:** 2021-07-13

**Authors:** Charlotte Hayden, Jedd Raidan, Jonathan Rees, Abhishek Oswal

**Affiliations:** 1 Bristol Royal Infirmary, South Bristol Academy, University Hospitals of Bristol and Weston NHS Foundation Trust, Upper Maudlin Street, BS2 8HW Bristol, UK; 2 University of Bristol Medical School, 5 Tyndall Avenue, BS8 1UD Bristol, UK

**Keywords:** Acute care, Clinical teaching, Workplace learning

## Abstract

**Background:**

New medical graduates are significantly unprepared to manage acutely unwell patients due to limited first-hand clinical exposure in the undergraduate curriculum. Supporting undergraduate learning in the acute setting can be challenging for junior doctors when balancing teaching and clinical responsibilities. Our aim was to explore junior doctors’ first-hand experiences of supporting undergraduate education in the acute admissions environment(take).

**Methods:**

Fourteen junior doctors in one teaching hospital in South West England took part in semi-structured focus groups (4–6 participants in each) which were audio-recorded, transcribed, and thematically analysed.

**Results:**

Junior doctors described their educational role as comprising: teaching, demonstrating, coaching, and supervising. They perceived the acute take as a highly variable, unpredictable setting that offered a broad scope for learning. Tensions between doctors’ clinical and educational roles were described, influenced by internal and external factors. Clinical work was prioritised over teaching and participants lacked confidence in supervisory and clinical skills. Doctors felt pressured to meet students’ expectations and lacked understanding of their educational needs. Senior colleagues were highly influential in establishing an educational culture and were often a source of pressure to deliver timely clinical care. Organisations were perceived not to value teaching due to the lack of provision of dedicated teaching time and prioritisation of limited resources towards patient care. Participants managed tensions by attempting to formally separate roles, demoting students to passive observers, and they sought greater continuity in placements to better understand students’ abilities and expectations.

**Conclusions:**

Educational opportunities for undergraduate students on the acute take are varied and highly valuable. This study provides insight into the provision of workplace education and its challenges from junior doctors’ perspectives. We highlight areas for improvement of relevance to educational providers.

## Background

Acute and emergency care is a dynamic, fast-paced environment, treating increasing numbers of complex patients with a wide range of conditions [1, 2]. This presents challenges both to the provision of care and education [2–5]. New medical graduates feel unprepared to work in the acute environment, citing limited exposure to acute care [6–8] and describe current teaching delivery as too theoretical with a lack of practical, clinical-based teaching [6, 9].

Early clinical experience and shadowing opportunities positively impact students’ feelings of preparedness for future practice [10]. Furthermore, students that actively interact with the clinical environment whilst on clinical placements feel better equipped to manage unwell patients [11, 12]. Educational theorists recognise the need for learners to contextualise classroom-based learning through active participation in the workplace [13–15] and the key role of clinicians in supporting engagement and facilitating learning [16].

Whilst many of the learning opportunities in the workplace are opportunistic and informal [17–21], medical students may expect more structured teaching [22]. Clinicians are expected to provide supportive yet challenging educational experiences [16] whilst balancing responsibilities of intensive, timely clinical care and ensuring patient safety and flow [13, 22, 23]. Junior doctors enjoy teaching and generally perceive themselves as knowledgeable and competent clinical teachers [24], however finding the time to teach students effectively in a busy clinical environment is challenging [14, 25]. Managing competing clinical and educational pressures may compromise the nature of the student-teacher interactions which in turn, may affect learning experiences. Positive interactions are constructive for student engagement [23] whilst negative interactions are shown to hinder learning[14, 25].

Whilst there is significant literature exploring student experiences of acute care placements, there is a lack of evidence of junior doctors’ perceptions of teaching in this setting. Our study aimed to explore junior doctors’ experiences of teaching undergraduates on the acute take: what is currently being taught, how it is taught, barriers to teaching and sought to identify areas for possible improvement.

## Methods

### Setting and participants

Clinical teaching fellows and junior doctors of FY2 grade and above were invited to take part in this study by email. All participants had expressed an interest in medical education and were based in one teaching hospital in the South West of England. Doctors at FY1 level were excluded as they had been in post for less than four weeks at the time of the study and therefore had limited experience of clinical teaching.

### Data collection

Participants took part in a focus group facilitated by one researcher (CH) who was working as a clinical teaching fellow in the same hospital. Focus groups were semi-structured based on the topic guide in Table 1, audio-recorded and subsequently transcribed verbatim.

**Table 1:** Focus group topic guide

Focus Group Topic Guide	
1. How much do you teach on the acute take? 2. How easy is it to teach on the acute take? 3. What kind of teaching do you deliver on the acute take? 4. What do you believe students need to learn from being on the acute take? 5. What are the barriers to teaching on the acute take? 6. How could you improve your teaching on the acute take?	

The term ‘teaching’ was used throughout the focus groups to encompass concepts such as supervision and coaching alongside more didactic teaching, as it was felt to be most relatable to junior doctors.

### Data analysis

Data were analysed thematically as defined by Braun and Clarke [24] using NVivo® software (QSR International, Massachusetts, USA) [25]. Transcriptions were read and independently coded by two researchers (CH and AO) before overarching themes were sought by CH. A final thematic structure and hierarchy were reviewed, defined, and named collaboratively through discussion between CH and AO. Data collection was limited by the number of respondents, though many themes reached data saturation.

### Ethics

Ethical approval for this study was granted from the University of Bristol Ethics Committee (Application ID 93362) on 16th August 2019. Electronic written consent was provided by participants before taking part in this study.

## Results

### Participants

Of the 26 doctors invited to take part, 12 did not respond or declined to participate. The 14 doctors who took part ranged from FY2 to ST3 (equivalent) level and worked in a broad range of inpatient specialties across medicine, surgery, paediatrics, emergency, and critical care (Table 2). Of these, ten were working in a split clinical and educational role. Three focus groups were carried out, each with four to six participants and lasting around 20 min.

**Table 2:** Participant demographics. *For those participants not in a training job, grade is presented as equivalent training grade. For reference: FY2 = 1–2 years postgraduate experience, ST1 = 2–3 years, ST2 = 3–4 years, ST3 = 4–5 years. †For those participants in part-time education roles, educational component ranged from 0.5–0.8 full-time equivalent

Participant	Grade*	Specialty	Current role^†^
1	ST1	Surgery: general and speciality	Full-time clinical role
2	ST2	Medicine: general or acute specialty	Part-time education role
3	ST2	Medicine: general or acute specialty	Part-time education role
4	ST3	Emergency and critical care	Part-time education role
5	ST1	Medicine: general or acute specialty	Part-time education role
6	FY2	Surgery: general and speciality	Full-time clinical role
7	ST1	Surgery: general and speciality	Part-time education role
8	FY2	Surgery: general and speciality	Full-time clinical role
9	ST1	Paediatrics	Part-time education role
10	ST1	Medicine: general or acute specialty	Part-time education role
11	ST1	Emergency and critical care	Part-time education role
12	FY2	Emergency and critical care	Full-time clinical role
13	ST3	Medicine: other specialty	Part-time education role
14	ST1	Medicine: general or acute specialty	Part-time education role

### Key themes

**Fig. 1:**
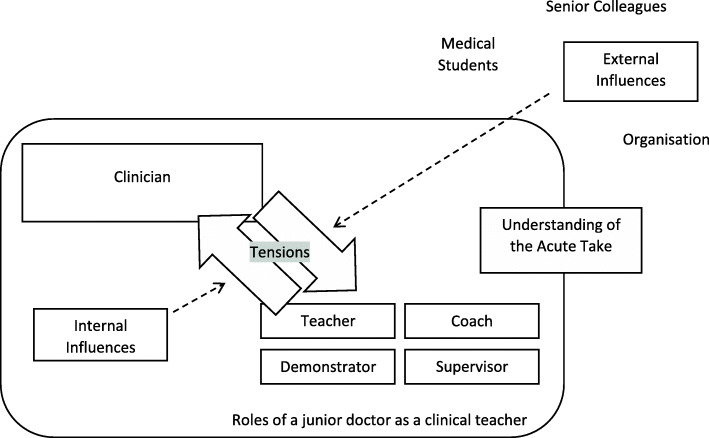
Schematic representation of key themes identified from focus groups

Junior doctors described various aspects of their role as clinical teachers and varied understanding of the acute take as a learning environment. Key sources of tension were identified in the integration of clinical and educational roles and attributed to either internal or external influences. Current strategies for managing these tensions were described by participants. Key themes are summarised in Fig. 1.

### Roles of the junior doctor as a clinical teacher

Participants described their clinical role as their primary identity; clinical responsibilities were perceived as separate to, and prioritised above, teaching responsibilities. When considering their educator identity, participants described four key aspects: teacher, demonstrator, coach, and supervisor, further detailed in Table 3.

**Table 3:** Four aspects of junior doctors’ educational role with description and illustrative quotes. *ACS = acute coronary syndrome. †Trop = troponin, biochemical marker of myocardial ischaemia.

Role	Description	Utility and impact	Example quote
**Teacher**	Didactic information delivery, discussion, or direct questioning.	High effort and interruptive to clinical practice, low to moderate yield for student learning. Used when clinical pressures allowed.	*“there’s probably less [sic] than ten topics they want to know about, you can just take them to one side and teach them a little bit” – P7 (FG2)* *“I’ve often just talked verbally at whoever was following me or shadowing me […] I’m not sure what they gain from it” - P12 (FG3)*
**Demonstrator**	Supporting student through active or passive observation of clinical practice.	Low effort for junior doctors, but low impact on students’ learning. Adopted more frequently for unwell patients.	*“or if [the patient] was sick, I’d ask [the student] to join me” - P13 (FG3)* *“they’re just, like ‘oh can I shadow you?’ and, you know, you don’t really want to watch me doing discharge summaries and stuff” – P7 (FG2)* *“some of them literally do just want to follow you around, which – fine! – [but] I would say is less effective in teaching” - P12 (FG3)*
**Coach**	Direct observation of skills, actual or simulated clinical practice with feedback.	Time intensive for junior doctors, but high yield learning for students. Rarely used on the acute take.	*“Prioritisation. Though that’s quite a difficult thing to teach […] it’s quite hard [for them] to do that unless they’re sort of with you […] you don’t have time for that.” - P4 (FG1)* *“[referring to teaching that is time consuming] I think bedside teaching because you have to go there and be with them and they’ll do half an hour of work and examinations, that’s just not going to work on take” - P5 (FG1)*
**Supervisor**	Supporting independent clinical practice and active participation.	Low effort for junior doctors, highly effective learning experience for students when managed well. Used for more experienced students and with suitable patients of lower clinical acuity.	*“they’re normally quite good about being independent and going and seeing somebody and feeding back” – P1 (FG1)* *“[referring to hands-on clinical experience] by the time they’re fifth year I think learning through doing those type of things is actually useful for them” – P11 (FG3)* “you know the ‘query ACS’* – they’ve already been clerked, they’ve had their bloods taken and you’re awaiting another trop†, that’s a perfect opportunity for the students to go [and see them]” – P13 (FG3)

### Understanding of the acute take as a learning environment

Participants recognised the acute take as a highly variable environment with significant fluctuations in clinical demand. At busy times, it was challenging to adopt more time-intensive teaching methods and participants felt students’ learning experiences suffered.

*“I mean there were busy days, but I would say more often we would have time to actually […] for them to clerk someone and then to actually go through it whereas on the medical take it was difficult.” – P9 (FG2)*.

Whilst at less busy times doctors felt they could deliver more student-specific, didactic teaching, they recognised that the lack of authentic patient-centred educational opportunities could mean students had a less valuable experience.

*“on surgery that we’re actually quite lucky in a sense that actually sometimes it’s quite quiet […] you feel a bit like they’ve been, kind-of, left short at the end of it?” – P1 (FG1)*.

*“it’s slightly pot-luck as to whether something that’s of educational value happens to happen that day or when that happens” – P9 (FG2)*.

Conceptually straightforward, ‘textbook’ clinical cases were perceived to be easier learning opportunities and such cases were more prevalent in some specialties than others. The variety and breadth of patient presentations made targeting teaching a challenge, though some specialties were described as having fewer core presentations that could be taught by junior doctors.

*“medical patients are more complex […] it’s rarely a cut and dry pneumonia […] in surgery it’s RIF pain - they all get fluids, antibiotics, analgesia and kind of refer upwards.” - P10 (FG2)*.

*“the acute medical take has such a ferocious range of things that you could see that it’s very hard to target things, whereas, in the specialties where there are far fewer presenting complaints, the junior doctor might be much more confident to teach at FY1 or FY2 level.” - P10 (FG2)*.

Junior doctors recognised the broad range of learning opportunities available on the acute take and participants agreed that student attachments were highly valuable.

*“we’d go through x-rays, ECGs, gases, differentials, more resource-based than actually at the bedside, unless someone had something really interesting, I was like ‘oh come and look at this sign’. Yeah. Kind of bits around [the case].” – P4 (FG1)*.

### Tensions between clinical and educational roles

#### Internal influences

Junior doctors overwhelmingly perceived their clinical role to be more important than educational roles and as a result clinical work was often seen as a barrier to teaching.

*“Time pressures, guilt. […] it slows you down a lot if you have a student with you for a whole clerking and you’re having to explain your work” – P2 (FG1)*.

*“you’re trying to manage a busy take, often, and trying to be as efficient as you can and then you’re consciously [sic], you’re conscious of the amount of people that are waiting” – P11 (FG3)*.

Participants lacked confidence in their abilities both as educators and clinicians. They perceived their lack of specialist knowledge (gained through postgraduate membership exams) and reliance on senior colleagues for clinical decision-making precluded them from delivering high-quality teaching to students.

*“I might not know myself if it’s a really complex patient, and [I’d] want to discuss it with someone else […] that also creates a bit of a barrier to teaching as well if you’re not sure yourself” – P8 (FG2)*.

Participants recognised the value of experiential learning through active participation, though their lack of confidence often led to anxiety when taking responsibility for the student.

*“forcing them to think rather than be passive listeners […] if you get them to clerk on the acute take or get them to go and look things up on the acute take I think it’s much more real and relevant and stimulating.” – P10 (FG2)*.

*“I’m not so further [sic] on in my career […] I’d find it very difficult to have someone I was worried about with a student […] I can’t do the teaching alongside dealing with someone who’s that unwell.” – P6 (FG1)*.

#### External influences

There was a lack of understanding of students’ learning requirements. Despite this, participants perceived pressure from students to deliver targeted, relevant teaching to individual students’ needs, even when students did not recognise what these were.

*“I found it really difficult actually with balancing […] either: do they want knowledge or do they want to know the practical elements of the job?” – P8 (FG2)*.

*“It was like an extra pressure to try and make sure that the students were getting what they wanted” - P7 (FG2)*.

The unpredictable nature of the acute take and fluctuating numbers of patients made meeting students’ needs even more challenging. Junior doctors recognised that whilst it was important for students to have clear aims these should not be too specific.

*“That’s the difficulty isn’t it […] they want to be taught X but there’s nothing, ‘we’ve got no X today, we’ve only got Y and Z’ so that’s quite difficult, so they have to have quite broad goals, but they need to have something they want from it” - P4 (FG1)*.

Some participants noted that, when students had communicated clear aims of their clinical attachment, they perceived it as box-ticking. This led to perceptions that students were poorly engaged and not committed to staying the full length of their allocated shift.

*“they’re sticking around for less than four hours though, maybe one or two? They’ll sort of have an expectation that they’ll go and clerk a patient and then they’ll want to present to you then they’ll be done” - P12 (FG3)*.

There were broader perceptions of students’ lack of engagement due to the variability of student presence on the acute take. Participants also noted that students often did not attend shifts when they were scheduled to.

*“it’s rare to have a student and it’ll probably be the first day, or the first or second day of their new block and after that they just don’t turn up again.” - P1 (FG1)*.

*“the other limit that I sort of, you know, no one really talks about is the students just don’t turn up”- P10 (FG2)*.

Senior doctors were felt to be highly influential in determining the culture of teaching on the acute take. Sometimes these colleagues reinforced participants’ beliefs that teaching was a secondary activity and clinical work should be prioritised at all costs, whereas others (particularly those more involved with formal teaching delivery) were felt to be positively influential.

*“[referring to consultants] I am aware that sometimes people are less keen for you and they do kind of, hound a bit and be like, not ‘stop teaching’, but ‘you need to prioritise a bit differently’.” – P4 (FG1)*.

*“I think the general principle is when the consultants have more involvement in the academies or more involvement in teaching, in general, they’re going to be more engaging and they’re going to promote it more.” – P11 (FG3)*.

Some participants explicitly described this organisational culture enacted by senior clinicians. They added that the lack of provision of protected teaching time by organisations suggested they did not support doctors delivering clinical teaching.

*“There is a culture that comes with the NHS, it is top-down and you kind of feel you have to abide by that, […] you have to go with what the culture is at the time on that take” - P12 (FG3)*.

*“acknowledgement from the organisation that you’re going to have students attached to the take then […]” “ they need to give you time to help the students” - P2 and P4 (FG1)*.

Senior colleagues were also regarded as a constant pressure to deliver timely clinical care, particularly when faced with staffing shortages or high waiting times. Junior doctors were aware of the scarcity of organisational resources such as staffing, computers, and physical space. They described a tendency for resources to be protected for patient care and as a result, side-lining education.

*“The presence of other doctors watching your workload makes a huge difference” - P7 (FG2)*.

*“you get an impression from consultants who have expected you to have seen a certain amount of patients by the time they come and post-take with you and you do get a bit of pressure” - P11 (FG3)*.

*“if your staffing levels are low and waiting times are high then you don’t want to slow down” - P14 (FG3)*.

*“you often wouldn’t be able to get to a computer to order your investigation let alone get up resources for them to learn from so…that was a big problem” – P11 (FG3)*.

### Managing tensions

 To manage tensions in fulfilling both clinical and teaching responsibilities, participants attempted to formally compartmentalise clinical and educational roles through breaking up what they perceived to be ‘teaching time’ into small sections and delivering this in-between clinical care.

*“It’s just ‘look at this ECG – there’s a finding on it – I’ll just teach you about that for two minutes’ then crack on” - P4 (FG1)*.

This participant suggested that protected time built into their job plan would help avoid the pressures of balancing clinical and educational responsibilities.

*“Allocated time would be helpful. You could have a half-hour slot or something where you can say ‘I’m taking myself out of the take, this is going to be purely education’” - P4 (FG1)*.

As a result of anxieties around students’ clinical competence and participants’ concerns of patient acuity, students were often relegated to observer roles rather than encouraged to see patients independently. This was particularly common with more acutely unwell patients.

*“I sometimes feel nervous about the acuity of the patients as well, so if you’re going to put your name next to that person, you’re taking responsibility. […] quite often I’d just be like ‘we’ll talk about it afterwards’ because I don’t feel like, I don’t, I can’t do the teaching alongside dealing with someone who’s that unwell” – P6 (FG1)*.

Participants recognised a need to better understand students’ abilities and expectations to reconcile their doubts about students’ competence and ensure they met students’ expectations. They felt this was best achieved through greater continuity in students’ placement within the team.

*“without sounding falsely nostalgic, the loss of the firm structure […] you’d get to know [the junior doctors] and they would be able to teach you stuff much more consistently and build on the previous teaching sessions.” - P10 (FG2)*.

## Discussion

### Key findings and relation to literature

Junior doctors describe a clear separation between clinical and educational roles and consistently feel their clinical responsibilities outweighed their educational ones. This hierarchical identity placement leads to tensions [26–28] which are reinforced or challenged by students, senior colleagues, and organisations.

Participants’ perceptions that intense clinical work becomes a barrier to teaching likely influences their adoption of low effort and less time-intensive teaching styles. Coaching and supervision are rated by students as the most effective clinical teaching strategies [29, 30] but are time-intensive and challenging for junior doctors to deliver in the context of high clinical demand and varied patient presentations [5, 31].

Whilst junior doctors are effective near-peer tutors in other settings [32], they perceive themselves as novice teachers which makes workplace supervision and supporting student participation particularly challenging [33–35]. Lack of confidence in managing acutely ill patients is a common perception amongst newly qualified doctors [6–10] and is seen to exacerbate these feelings. Ultimately, achieving a coherent identity as a clinical teacher [26] is difficult for junior doctors when they do not recognise themselves as competent teachers or clinicians.

Current strategies to manage tensions, such as compartmentalising roles and reducing student participation in response to anxieties, are likely to impair students’ authentic interactions with the workplace[26]. The importance of student engagement in active, well-supported, experiential learning is well recognised [14, 16, 36] and our findings suggest a lack of support for junior clinical teachers to facilitate this.

Participants suggest that continuity of attachment to clinical teams might mitigate anxieties around supervising students. Such strategies have been shown to help meet students’ individual needs and promote enthusiasm for teaching [37]. Placements that acknowledge and develop student responsibility, such as assistantships, also promote students’ feelings of preparedness [38, 39]. Formalised teacher training for newly qualified doctors may support the development of clinical teaching skills [40, 41] and improving awareness of local curricula may aid delivery of relevant teaching. Organisations that support training in teaching could help form an institution-wide culture where teaching is valued[42].

This study demonstrates junior doctors’ awareness of teaching cultures rooted within an organisation and amongst senior team members [14]. Professional regulators have appealed to local service providers to recognise and foster an educational culture [43]. Our participants suggest organisational support for teaching on the acute take does not currently go far enough; they back others’ calls for teaching time to be built into their job plan [44]. Other research recognises the positive influence of enthusiastic and welcoming senior clinicians [4, 23, 45] and these findings reinforce the need for such individuals to support junior doctors in the acute setting.

Finally, our findings suggest organisations must be cautious in balancing the rhetoric of clinical pressures and patient safety with that of delivering educational responsibilities, in order to ease tensions that junior doctors experience. Others have echoed participants’ concerns that quality of teaching suffers in response to high workload [32, 41], but evidence suggests students still value placements in busy clinical environments [8, 46]. Other research has assured that student satisfaction from placements remains positive even when the workload is higher [4, 47].

Acute clinical placements are vitally important in developing the necessary skills for competent practice in the admissions environment [6, 9, 10]. There are currently significant deficiencies in undergraduate clinical training resulting in junior doctors who are underprepared to manage acutely unwell patients. These findings ultimately highlight the value of investing in workplace education to foster capable doctors who will go on to become more confident clinical teachers.

### Strengths and limitations

This study presents a rich narrative, offering novel perspectives, with participants drawing on their teaching experiences across a range of specialties and hospitals, all underpinned by their own learning experiences. With a peer facilitator, participants’ views are likely to have been explored in a more relaxed and non-judgemental setting than with an unfamiliar, non-expert facilitator. Results are likely to offer a more in-depth understanding, though this approach risks researchers’ conscious and subconscious biases, especially during data interpretation and analysis, however we offer a degree of reflexivity in our conclusions [48].

Participants in part-educational, part-clinical roles are actively engaged with local medical education provision, and are assumed to have some understanding of formal teaching theories and methods, though this may be limited as the study was conducted within six weeks of commencing their placements. Purposive sampling means we lack insight from those less inclined to teach, nor do we gain much appreciation of any significant barriers to engagement in undergraduate clinical teaching.

### Implications for practice

Three key opportunities for improvement were identified. Firstly, supporting junior doctors to develop an integrated clinical and educational identity. This may include greater recognition of educational involvement by senior doctors and organisations for example through dedicated teaching time ‘on the job’. Secondly, we note that both students and junior doctors need a better, shared understanding of general and student-specific aims and objectives. Both could be achieved through improving junior doctors’ training in teaching methods with particular focus on supervision and local curricula.

Finally, organisations should work collaboratively with educational providers to facilitate a more independent, active role for medical students on the acute take allowing for limited facilitation by junior doctors. Appropriate resources should be allocated for this, accounting for the breadth and variability of opportunities that present. Greater continuity and consistency of supervision on acute care placements may improve the development of supportive educational relationships between junior doctors and medical students.

## Conclusions

The acute admissions environment presents myriad opportunities for undergraduate learners and many challenges for junior doctors in managing responsibilities of patient care with clinical teaching. This exploratory study suggests key areas for targeted improvement to the delivery of near-peer clinical education through highlighting learning opportunities for students’ supported participation and fostering junior clinicians’ teaching skills with the provision of necessary resources. Through this insight, we hope to maximise learning yield from authentic experiences and encourage the development of a supportive culture for clinical teaching and undergraduate education within a busy, but rich learning environment.

## Data Availability

The data obtained during this study are available from the corresponding author upon reasonable request.

## Abbreviations

ACS: Acute coronary syndrome
ECG: Electrocardiogram
FG1, FG2 etc: Focus Group 1, Focus Group 2 etc
FY1: Foundation Year One (Doctor)
FY2: Foundation Year Two (Doctor)
NHS: National Health Service
P1, P2 etc: Participant 1, Participant 2 etc
ST1: Specialty Trainee Year 1
ST2: Specialty Trainee Year 2
ST3: Specialty Trainee Year 3
